# A year in pharmacology: new drugs approved by the US Food and Drug Administration in 2020

**DOI:** 10.1007/s00210-021-02085-3

**Published:** 2021-04-16

**Authors:** Gizem Kayki-Mutlu, Martin C. Michel

**Affiliations:** 1 Department of Pharmacology, Faculty of Pharmacy, Ankara University, Ankara, Turkey; 2 Department of Pharmacology, Johannes Gutenberg University, Langenbeckstr. 1, 55118 Mainz, Germany

**Keywords:** FDA, New drugs, First-in-indication, First-in-class

## Abstract

While the COVID-19 pandemic also affected the work of regulatory authorities, the US Food and Drug Administration approved a total of 53 new drugs in 2020, one of the highest numbers in the past decades. Most newly approved drugs related to oncology (34%) and neurology (15%). We discuss these new drugs by level of innovation they provide, i.e., first to treat a condition, first using a novel mechanisms of action, and “others.” Six drugs were first in indication, 15 first using a novel mechanism of action, and 32 other. This includes many drugs for the treatment of orphan indications and some for the treatment of tropical diseases previously neglected for commercial reasons. Small molecules continue to dominate new drug approvals, followed by antibodies. Of note, newly approved drugs also included small-interfering RNAs and antisense oligonucleotides. These data show that the trend for declines in drug discovery and development has clearly been broken.

## Introduction

In the 2020, the US Food and Drug Administration (FDA) approved a total of 53 new drugs (https://www.fda.gov/drugs/new-drugs-fda-cders-new-molecular-entities-and-new-therapeutic-biological-products/novel-drug-approvals-2020), which is one of the highest numbers per year in the past two decades (Batta et al. 2020). This article reviews the degree of innovation reflected in these approvals. We consider a newly approved treatment for an indication where no treatment had previously been approved to be the highest level of innovation (“first in indication”). We consider first-in-class treatments as the second highest level. These provide treatments based on a novel mechanism of action for conditions for which other treatments had already been approved. The lowest level of innovation is considered for treatments that use the same mechanism of action as previously approved drugs in this indication. However, a lower level of innovation does not necessarily imply smaller clinical benefit as new compounds within a drug class may differ from others in efficacy, tolerability, and/or patient convenience to a medically meaningful extent. Discussing specific advantages and disadvantages of individual compounds is beyond the scope of this article. Based on these data, we discuss emerging trends in drug approvals.

## Methods

Our analysis is based on the list of new molecular entities approved by the FDA in 2020 as communicated by the agency (https://www.fda.gov/drugs/new-drugs-fda-cders-new-molecular-entities-and-new-therapeutic-biological-products/novel-drug-approvals-2020). Our analyses do not include vaccines, generics, or biosimilars, newly approved drug combinations unless at least one of the combination partners is a novel chemical or biological entity or already approved drugs that received approval for an additional indication and/or in a novel formulation. Other regulatory agencies may have approved the same compounds earlier than the FDA, may do so at later points in time, may choose not to approve some of these compounds, or may choose to approve compounds not approved by the FDA. These differences may at least partly reflect that originator companies may not have filed for approval in all jurisdictions, at least not at the same time. Our focus on FDA approvals does not imply any opinion on approvals by the FDA as compared to the regulatory authorities in other jurisdictions but rather uses the FDA as one of the most influential regulatory authorities as point of reference.

We have retrieved information on the indication, classified the innovation status (Table 1) and the type of agent such as small molecule, antibody, or other biological (Table 2). Where available, at least one key reference on pivotal clinical evidence for each newly approved molecular entity is provided for further reading. Confirming previously reported trends (Köster et al. 2016b; Köster et al. 2016a), we found that the disclosure of data in the peer-reviewed literature is very heterogeneous among compounds ranging from 0 in one case to several hundred in others. The subsequent discussion is based on therapeutic areas (Table 3).

**Table 1:** Newly approved drugs grouped by novelty

1st in indication		1st in class		Other	
Drug	Major indication	Drug	Major indication	Drug	Major indication
Atoltivimab, maftivimab, odesivimab-ebgn	Ebola	Abametapir	Lice	Artesunate	Malaria
Lonafarnib	Premature aging	Belantamab; mafodotin-blmf	Multiple myeloma	Amisulpride	Postoperative nausea
Remdesivir	COVID-19	Bempedoic acid	High LDL cholesterol	Ansuvimab-zykl	Ebola
Setmelanotide	Pro-opiomelanocortin deficiency	Clascoterone	Acne	Avapritinib	Gastrointestinal stromal tumor
Tazemetostat	Epithelioid sarcoma	Flortaucipir f 18	Diagnostic agent for alzheimer’s disease	Berotralstat	Hereditary angioedema
Teprotumumab-trbw	Thyroid eye disease	Fostemsavir	HIV	Capmatinib	Non-small cell lung cancer
		Gallium 68 psma-11	Detect antigen positive prostate cancer	Cedazuridine, decitabine	Myelodysplastic sydrome
		Inebilizumab-cdon	Neuromyelitis optica spectrum disorder	Copper cu 64 dotatate	Detect neuroendocrine tumors
		Lumasiran	Hyperoxaluria type 1	Eptinezumab-jjmr	Migraine
		Osilodrostat	Cushing’s disease	Fluoroestradiol f 18	Detect receptor-positive breast cancer
		Sacituzumab govitecan-hziy	Triple-negative breast cancer	Isatuximab-irfc	Multiple myeloma
		Selumetinib	Neurofibromatosis type 1	Lactitol	Chronic idiopathic constipation
		Satralizumab-mwge	Neuromyelitis optica spectrum disorder	Lurbinectedin	Small cell lung cancer
		Tafasitamab-cxix	Large B cell lymphoma	Margetuximab-cmkb	Her2+ breast cancer
		Tirbanibulin	Actinic keratosis	Naxitamab-gqgk	Neuroblastoma
				Nifurtimox	Chagas disease
				Oliceridine	Acute pain
				Opicapone	“Off” episodes of Parkinson’s disease
				Ozanimod	Multiple sclerosis
				Pemigatinib	Cholangiocarcinoma
				Pralsetinib	Non-small lung cancer
				Relugolix	Prostate cancer
				Remimazolam	For sedation
				Rimegepant	Migraine
				Ripretinib	Gastrointestinal-stromal tumors
				Risdiplam	Spinal muscular atrophy
				Selpercatinib	Lung and thyroid cancers
				Somapacitan-beco	Growth hormone deficiency
				Triheptanoin	Long-chain fatty acid oxidation disorders
				Tucatinib	HER2-positive breast cancer
				Vibegron	Overactive bladder
				Viltolarsen	Duchenne muscular dystrophy

For definitions, see the “Introduction”

**Table 2:** Newly approved drugs by drug type

Small molecule		Antibody		Gene-based		Peptide	
Drug	Major indication	Drug	Major indication	Drug	Major indication	Drug	Major indication
Abametapir	Lice	Ansuvimab-zykl	Ebola	Lumasiran	Hyperoxaluria type 1	Somapacitan-beco	Growth hormone deficiency
Amisulpride	Postoperative nausea	Atoltivimab, maftivimab, odesivimab-ebgn	Ebola	Viltolarsen	Duchenne muscular dystrophy		
Artesunate	Malaria	Belantamab, mafodotin-blmf	Multiple myeloma				
Avapritinib	Gastrointestinal stromal tumor	Eptinezumab-jjmr	Migraine				
Bempedoic acid	High LDL cholesterol	Inebilizumab-cdon	Neuromyelitis optica spectrum disorder				
Berotralstat	Hereditary angioedema	Isatuximab-irfc	Multiple myeloma				
Capmatinib	Non-small cell lung cancer	Margetuximab-cmkb	Her2+ breast cancer				
Cedazuridine, decitabine	Myelodysplastic syndrome	Naxitamab-gqgk	Neuroblastoma				
Clascoterone	Acne	Sacituzumab govitecan-hziy	Triple-negative breast cancer				
Copper Cu 64 dotatate	Detect neuroendocrine tumors	Satralizumab-mwge	Neuromyelitis optica spectrum disorder				
Flortaucipir f 18	Diagnostic agent for Alzheimer’s disease	Tafasitamab-cxix	Large B cell lymphoma				
Fluoroestradiol f 18	Detect receptor-positive breast cancer	Teprotumumab-trbw	Thyroid eye disease				
Fostemsavir	HIV						
Gallium 68 psma-11	Detect antigen positive prostate cancer						
Lactitol	Chronic idiopathic constipation						
Lonafarnib	Premature aging						
lurbinectedin	Small cell lung cancer						
Nifurtimox	Chagas disease						
Oliceridine	Acute pain						
Opicapone	“Off” episodes of Parkinson’s disease						
Osilodrostat	Cushing’s disease						
Ozanimod	Multiple sclerosis						
Pemigatinib	Cholangiocarcinoma						
Pralsetinib	Non-small lung cancer						
Relugolix	Prostate cancer						
Remdesivir	COVID-19						
Remimazolam	For sedation						
Rimegepant	Migraine						
Ripretinib	Gastrointestinal-stromal tumors						
Risdiplam	Spinal muscular atrophy						
Selpercatinib	Lung and thyroid cancers						
Selumetinib	Neurofibromatosis type 1						
Setmelanotide	Pro-opiomelanocortin deficiency						
Tazemetostat	Epithelioid sarcoma						
Tirbanibulin	Actinic keratosis						
Triheptanoin	Long-chain fatty acid oxidation disorders						
Tucatinib	HER2-positive breast cancer						
Vibegron	Overactive bladder

**Table 3:** Newly approved orphan drugs

Drug	Major indication
Ansuvimab-zykl	Ebola
Atoltivimab, maftivimab, and odesivimab-ebgn	Ebola
Avapritinib	Gastrointestinal stromal tumor
Belantamab mafodotin-blmf	Multiple myeloma
Berotralstat	Hereditary angioedema
Capmatinib	Non-small cell lung cancer
Cedazuridine, decitabine	myelodysplastic syndromes
Copper Cu 64 dotatate	To detect neuroendocrine tumors
Inebilizumab-cdon	Neuromyelitis optica spectrum disorder
Isatuximab-irfc	Multiple myeloma
Lonafarnib	Premature aging
Lumasiran	Hyperoxaluria type 1
Lurbinectedin	Small cell lung cancer
Naxitamab-gqgk	Neuroblastoma
Nifurtimox	Chagas disease
Osilodrostat	Cushing’s disease
Pemigatinib	Cholangiocarcinoma
Pralsetinib	Non-small lung cancer
Ripretinib	Gastrointestinal-stromal tumors
Risdiplam	Spinal muscular atrophy
Satralizumab-mwge	Neuromyelitis optica spectrum disorder
Selpercatinib	Lung and thyroid cancers
Selumetinib	Neurofibromatosis type 1
Setmelanotide	Pro-opiomelanocortin deficiency
Tafasitamab-cxix	Large B-cell lymphoma
Tazemetostat	Epithelioid sarcoma
Teprotumumab-trbw	Thyroid eye disease
Triheptanoin	Long-chain fatty acid oxidation disorders
Tucatinib	HER2-positive breast cancer
Viltolarsen	Duchenne muscular dystrophy

## Oncology

In 2020, cancer drugs dominated the list of newly FDA-approved drugs with 18 (34% of total) products in 2020 (Mullard 2021). The methyltransferase inhibitor, *tazemetostat* is the first therapy approved to treat adults and pediatric patients over 16 years old with epithelioid sarcoma that cannot be surgically removed. Epithelioid sarcoma is a rare soft tissue sarcoma occurring mostly in young adults. Dysregulation of histone methyltransferase Enhancer of Zeste Homolog 2 is known to play a critical role as an oncogenic factor in a variety of cancer types (Hoy 2020). Thus, Enhancer of Zeste Homolog 2 inhibition attracted attention as a potential therapeutic target. Tazemetostat caused a complete or partial tumor shrinkage with a 15% overall response rate in phase II clinical trials (Gounder et al. 2020), which led to an accelerated approval. The most frequently observed adverse events (AE) included not only pain, fatigue, nausea and vomiting, and loss of appetite but also hemorrhage, pleural effusion, skin infection, dyspnea, and respiratory distress. Phase III trials are still ongoing.

*Pemigatinib* is a fibroblast growth factor receptor inhibitor. It is the first targeted treatment approved for adults with advanced bile duct cancer (cholangiocarcinoma). A variety of genetic alterations are identified and among them particularly fibroblast growth factor receptor alterations are found in the patients with cholangiocarcinoma (Pellino et al. 2018). Pemigatinib has been demonstrated to achieve a partial or complete shrinkage with a 36% overall response rate (Abou-Alfa et al. 2020), followed by an accelerated approval. The most common AE was hyperphosphatemia along with hypophosphatemia and arthralgia (Abou-Alfa et al. 2020).

Three new therapies were approved for the treatment of various types of breast cancer. *Sacituzumab govitecan-hziy* was approved to treat metastatic triple-negative breast cancer. It is a trophoblast cell surface antigen 2-targeted antibody and topoisomerase inhibitor conjugate and a first-in-class antibody-drug conjugate. Clinical benefit rate was reported to be 45.4% with a median progression-free survival of 5.5 months and overall survival of 13.0 months (Bardia et al. 2019). It is given by intravenous (IV) infusion once a week on days 1 and 8 at 21-day treatment cycles. Anemia and neutropenia were the most common AE reported (Bardia et al. 2019). For the treatment of metastatic human epidermal growth factor receptor (HER)2-positive breast cancer, a kinase inhibitor *tucatinib* (Shah et al. 2021) and a HER2/neu receptor antagonist *margetuximab* (Rugo et al. 2021) were approved. Adding tucatinib to other medications (trastuzumab and capecitabine) resulted in greater progression-free survival and overall survival but increases the risk of diarrhea and elevated aminotransferase levels (Murthy et al. 2019). A clinical trial comparing margetuximab with trastuzumab showed a significant improvement in progression-free survival in patients treated with margetuximab (Rugo et al. 2021). The main serious complication associated with its use was ventricular dysfunction (Rugo et al. 2021). Tucatinib is the first new drug approved under international collaboration and both tucatinib and margetuximab were granted orphan drug designation.

Two kinase inhibitors, *capmatinib* and *pralsetinib* were approved for the treatment of metastatic non-small cell lung cancer. Capmatinib (Alzofon and Jimeno 2021) is the first therapy approved against specific mutations that lead to mesenchymal-epithelial transition or MET exon 14 skipping. Sixty-eight percent of the patients who have not been previously treated and 41% of who received a previous medication experienced complete or partial shrinkage of their tumors. Peripheral edema was the most frequently observed AE (Wolf et al. 2020). Another kinase inhibitor, *selpercatinib* (Solomon et al. 2021) was approved for the treatment of metastatic non-small cell lung cancer, medullary thyroid cancer, and other types of thyroid cancers. It is the first treatment approved especially for patients with a RET gene alteration. Selpercatinib treatment achieved an antitumor activity with an overall response rate of 64%. The most common AE were increased AST and ALT, hypertension, and dry mouth (Goto et al. 2020). For the treatment of metastatic small cell lung cancer, an alkylating drug, *lurbinectedin* was approved to be used during or after platinum-containing therapy. It was reported to mediate a tumor shrinkage that lasted more than 6 months in the 35% patients and hematological abnormalities were reported as the common AE (Trigo et al. 2020).

A gonadotropin-releasing hormone receptor antagonist, *relugolix* (Shore et al. 2020) was approved as the first oral therapy to treat advanced prostate cancer. Of the patients treated with relugolix, 96.7% maintained castration-level androgens for 48 weeks, along with a lower risk of cardiovascular events compared with leuprolide-treated group, although cardiac rhythm problems were still among the possible AE (Shore et al. 2020). Interestingly, relugolix is also under late-stage clinical investigation for the treatment of uterine fibroids (Rocca et al. 2020). *Tafasitamab-cxix* (Salles et al. 2020) is a CD19-directed cytolytic antibody that was approved under accelerated approval as a first-in-class medication. It is used for the treatment of the patients with diffuse large B cell lymphoma, which is a fast-growing cancer of the lymphatic system. It is administered through IV infusion in combination with lenalidomide. Tafasitamab treatment was shown to mediate a 55% complete or partial shrinkage lasting about 22 months. Pneumonia, febrile neutropenia, and pulmonary embolism were the most frequently observed serious AE (Salles et al. 2020).

For rare cancers such as multiple myeloma, two different antibody-based therapeutics were approved: CD38-directed cytolytic antibody, *isatuximab-irfc* and B cell maturation antigen-directed antibody, *belantamab mafodotin-blmf*. Isatuximab (Richardson et al. 2020) is used in combination with pomalidomide and dexamethasone and was shown to improve progression-free survival by 11.5 months. The most frequent AE were infusion reactions and upper respiratory tract infections (Attal et al. 2019). It was granted orphan drug designation and received its first approval in USA. Belantamab mafodotin-blmf (Lonial et al. 2020), on the other hand, was approved as a first-in-class medication. It mediates anti-myeloma activity and was shown to achieve an overall response of 31%. Keratopathy, thrombocytopenia and anaemia were the most common AE (Lonial et al. 2020). *Naxitamab-gqgk* (Markham 2021a) is a GD2-binding monoclonal antibody that is used to treat children 1 year of age and older and adults with neuroblastoma in bone or bone marrow. It was approved under accelerated approval. Naxitamab-gqgk is used in combination with a granulocyte-macrophage colony-stimulating factor that is given 5 days prior to naxitamab and continuing until the last day of the treatment. Naxitamab treatment was reported to mediate a complete or partial shrinkage in 45% of the patients (Kushner et al. 2018). Infusion-related reactions, pain, and tachycardia were the most common AE.

A nucleoside metabolic inhibitor, decitabine and a cytidine deaminase inhibitor, *cedazuridine* were approved as a combination for the treatment of myelodysplastic syndromes including chronic myelomonocytic leukemia (Thota et al. 2021). Twenty-one percent of the patients receiving cedazuridine treatment experienced complete response which lasted about 7.5 months (Garcia-Manero et al. 2020). Neutropenia, trombocytopenia, and febrile neutropenia are the most common AE.

Two new kinase inhibitors were approved for the treatment of gastrointestinal stromal tumor (Abraham et al. 2020) that originates in the stomach, bowel, or esophagus. *Avapritinib* (Vallilas et al. 2021) is the first treatment approved for gastrointestinal stromal tumor in patients with a platelet-derived growth factor receptor alpha gene mutation. 88% patients experienced complete or partial shrinkage of the tumor which lasted at least 6 months (Heinrich et al. 2020). The most common AE reported was anemia. *Ripretinib* (Blay et al. 2020) was also approved as the first new drug as a fourth-line treatment for gastrointestinal stromal tumor. Sixty-four percent of the patients who have previously been treated and 84% of the patients who had never treated before experienced complete or partial shrinkage. The most frequently observed AE were hypertension, increased liver enzymes, and hyponatremia (Drilon et al. 2020).

## Neurology

Drugs for neurological disorders took the second place with 8 (15%) approvals. A catechol-O-methyltransferase inhibitor, *opicapone* (Scott 2021) was approved as an adjunctive treatment to levodopa/carbidopa. It is a new therapy used in patients with Parkinson’s disease who are having “off” episodes in which patients’ symptoms are increased and their medications are not working. Two clinical trials demonstrated that opicapone mediated a reduction in mean daily off time (Ferreira et al. 2016; Lees et al. 2017). Dyskinesia, insomnia, and hypotension were the most common AE.

*Ozanimod* (Sun et al. 2020) is a sphingosine 1-phosphate receptor modulator that was approved for the treatment of patients with relapsing forms of multiple sclerosis including: clinically isolated syndrome, relapsing-remitting disease, and active secondary progressive disease. Two clinical trials reported that ozanimod had a lower relapse rate than interferon β-1a (Cohen et al. 2019; Comi et al. 2019). Most common AE were upper respiratory infections, increased liver enzymes, and hypertension.

Two new therapies acting on the calcitonin gene-related peptide or its receptor were approved for migraine. *Eptinezumab-jjmr* (Bhakta et al. 2021) is a monoclonal antibody against calcitonin gene-related peptide and used to prevent migraine in adults. It is administered through IV infusion every 3 months. Eptinezumab treatment was shown to reduce monthly migraine days over 12 weeks of treatment (Lipton et al. 2020), and administration every 12 weeks was reported to be associated with early and sustained preventive effects (Smith et al. 2020). Nasopharyngitis was the most common AE. *Rimegepant* (Bhakta et al. 2021), on the other hand, is the first and only calcitonin gene-related peptide receptor antagonist available as an orally disintegrating tablet. It is used for treatment of acute migraine with or without aura. Rimegepant was found to be superior to placebo for relieving pain and preventing bothersome symptoms (Croop et al. 2019). The most frequent AE are nausea and urinary tract infection.

*Remimazolam* (Sneyd and Rigby-Jones 2020) is a benzodiazepine used for medical procedures shorter than 30 min, e.g., colonoscopy to start and maintain sedation. It was shown to mediate faster recovery of neuropsychiatric function compared to placebo and midazolam in patients undergoing colonoscopy and bronchoscopy (Rex et al. 2018; Pastis et al. 2019). Hypotension and hypoxia may be observed after the use of remimazolam. There have also been new therapies approved for rare neurological diseases. *Risdiplam* (Baranello et al. 2021) is a selective survival of motor neuron-2 gene splicing modifier. It was approved to treat patients two months of age and older with spinal muscular atrophy that is a rare genetic disease in which muscle strength and the ability of movement is diminished, as the first oral drug. Forty-one percent of the patients were found to be able to sit independently more than 5 s after 12 months of treatment, and 81% of the patients did not need permanent ventilation after 23 or more months of treatment (Baranello et al. 2021). Most common AE are pneumonia, respiratory tract infection, and acute respiratory failure. *Viltolarsen* (Iftikhar et al. 2021) is an antisense oligonucleotide approved for the treatment of Duchenne muscular dystrophy (DMD) in patients with a specific DMD gene mutation. It is the second approved targeted treatment for this type of mutation. DMD is caused by low levels of dystrophin resulting in muscle weakness and premature death and it primarily affects boys. It is also under clinical investigation for spinal muscular atrophy. A major dystophin production was observed after 20–24 weeks of viltolarsen treatment (Iftikhar et al. 2021). Upper respiratory infections and injection site reactions are the most common AE.

A kinase inhibitor, *selumetinib* (Mukhopadhyay et al. 2021) is used for the treatment of plexiform neurofibroma that occurs in neurofibromatosis type 1 (NF1). It is the first therapy approved for pediatric patients over 2 years old. A partial response lasting more than 1 year was observed in 70% of the patients. The most common AE were nausea, vomiting, increase in the creatine phosphokinase level, skin rash and paronychia (Gross et al. 2020). Two antibody-based therapies were approved for the treatment of neuromyelitis optica spectrum disorder, a rare autoimmune disease that commonly affects optic nerves and spinal cord. *Inebilizumab-cdon* (Levy et al. 2021) is a CD19-directed cytolytic antibody and is the second approved therapy approved for this disorder, after eculizumab was approved in 2007. Antibody formation was shown in 213 of 230 patients, and attacks of the disease were reduced by 77% in antibody positive patients (Cree et al. 2019). Urinary tract infections, arthralgia, and infusion-related reactions are the most frequent AE. The third approved treatment for neuromyelitis optica spectrum disorder, *satralizumab-mwge* (Levy et al. 2021) is an interleukin-6 receptor antagonist. Two clinical trials reported that satralizumab treatment is effective for antibody formation which is associated with the decrease in the number of relapses (Yamamura et al. 2019; Traboulsee et al. 2020). The most frequent AE are flu-like symptoms, headache, upper respiratory tract infections, gastritis, and rash. A novel opioid agonist, *oliceridine* was approved for the treatment of acute severe pain in adults. Unlike other opioid agonists currently in use, oliceridine is selective to the G protein pathway, rather than for β-arrestin, thus may be associated with fewer AE (Markham 2020). Oliceridine treatment has been demonstrated to reduce pain after bunionectomy (Viscusi et al. 2019) and abdominoplasty (Singla et al. 2019). Gastrointestinal AE were observed in a dose-dependent manner.

### Metabolic and endocrine disorders

The FDA approved a treatment for weight management for the first time. The melanocortin 4 receptor agonist, setmelanotide (Clément et al. 2020) was approved for use in obese patients over 6 years old with specific enzyme deficiencies (proopiomelanocortin (POMC), proprotein convertase subtilisin/kexin type 1 (PCSK1), or leptin receptor (LEPR) deficiency). It is a daily subcutaneous injection. Leptin-melanocortin pathway plays an important role in the body weight regulation. Leptin produced from adipose tissue and binds its receptors, LEPR, on POMC-expressing neurons. During fed state, leptin activates POMC production that is processed by PCSK1 into melanocyte-stimulating hormone, which binds to MC4R leading to a reduced food intake (Yazdi et al. 2015). Some variants in genes affecting MC4 pathway leads to early-onset obesity (Clément et al. 2020). Clinical trials have reported that setmelanotide results in weight loss in patients with such genetic alterations, with 10% weight loss achieved in 80% of patients with POMC or PCSK1 deficiency and 46% of patients with LEPR deficiency (Clément et al. 2020). The most frequent AE are injection site reaction and hyperpigmentation, followed by nausea and vomiting. A human growth hormone analog, *somapacitan-beco* (Otsuka et al. 2020) was approved as a replacement therapy in patients with growth hormone deficiency. It is administered subcutaneously once a week, in contrast to previous therapies injected daily, thereby providing greater patient convenience. Patients receiving somapacitan have experienced a decrease in truncal fat percentage and improved parameters including visceral fat and lean body mass (Johannsson et al. 2020). The most frequent AE are back and joint, joint pain, indigestion, sleep problems, and decreased adrenal gland function. *Osilodrostat* (Rasool and Skinner 2021) that is a cortisol synthesis inhibitor, was approved for the treatment of Cushing’s disease. It is the first approved treatment addressing the cortisol overproduction by blocking 11-beta-hydroxylase. *Lumasiran* (Scott and Keam 2021), a HAO1-directed small interfering ribonucleic acid (siRNA), was approved as a first-in-class medication that is used to lower the level of urine oxalate in children and adults with primary hyperoxaluria type 1,which is a rare disease associated with oxalate accumulation. Lumasiran is the third approved siRNA treatment overall. Half of the patients receiving lumasiran had normal cortisol levels at the end of a 24-week treatment period and 86% of these patients maintained normal cortisol levels for 8 weeks. Most frequent AE were nausea, headache, fatigue, and adrenal insufficiency (Pivonello et al. 2020). An adenosine triphosphate-citrate lyase inhibitor, *bempedoic* acid (Nguyen et al. 2021) was approved as a first-in-class medication to treat high LDL cholesterol in patients with heterozygous familial hypercholesterolemia or with atherosclerotic cardiovascular disease. It is administered in tablet form in addition to diet and high-dose statin. Patients receiving bempedoic acid were shown to have lowered LDL-C levels along with the lowered non-high-density lipoprotein cholesterol, total cholesterol, apolipoprotein B, and high sensitivity C-reactive protein (Goldberg et al. 2019; Ray et al. 2019; Banach et al. 2020). Common AE include nasopharyngitis, urinary tract infection, and hyperuricemia. *Triheptanoin* (Hainque et al. 2019) is a medium-chain triglyceride that was approved for the treatment of long-chain fatty acid oxidation disorder. It is the only available therapy that provides calories and fatty acids to both pediatric and adult patients in whom muscle is broken down as a source of energy as fat cannot be used due to the enzyme deficiency. It is a liquid that is mixed with meals four or more times each day. Patients receiving triheptanoin were shown to have increased left ventricular ejection fraction and decrease LV wall mass. They also had lower heart rate during exercise compared with the patients receiving trioctanoin (Gillingham et al. 2017). Abdominal pain, diarrhea, vomiting, and nausea are the most frequent AE.

## Infectious diseases

COVID-19 has brought an unprecedented global challenge in 2020. Therefore, it is not surprising that the list of newly FDA-approved drugs included *remdesivir* (Beigel et al. 2020) as the first approved treatment of COVID-19 under an Emergency Use Authorization. It acts as a SARS-CoV-2 nucleotide analog and inhibits RNA polymerase of coronaviruses including SARS-CoV-2 that is the cause of COVID-19. It was approved for the treatment of both adult and pediatric patients over 12 years of age. Time to recovery was shorter while clinical improvement was higher in remdesivir-treated patients (Beigel et al. 2020). It has also been reported to improve patients’ clinical status (Goldman et al. 2020; Spinner et al. 2020). Nausea and increased liver enzymes are the most common AE. Investigation assessing the efficacy and safety in pediatric patients are still ongoing.

Several Zaire ebolavirus glycoprotein-directed human monoclonal antibodies were approved for the treatment of *Zaire ebolavirus* (Ebola virus) infection. Among them, *atoltivimab*, *maftivimab*, and *odesivimab-ebgn* (Inmazeb) were approved as a combination (REGN-EB3) therapy and considered the first FDA-approved therapy specifically for Ebola. It targets the glycoprotein on the surface of virus that helps the virus to fuse in the host cell. This combination of antibodies binds to this glycoprotein and blocks its attachment to the host (Markham 2021b). *Ansuvimab-zykl* (MAb114) is another antibody-based therapy approved for Ebola. They are both administered as an IV infusion and have been shown to lower mortality (Mulangu et al. 2019). Fever, chills, fast heart rate, fast breathing, and vomiting are the most frequent AE. Another antiviral approved is *fostemsavir*, a novel HIV-1 attachment inhibitor (Hiryak and Koren 2020). It was approved to treat HIV patients who cannot be treated with other therapies due to resistance. After an 8-day treatment, patients receiving fostemsavir had significantly decreased HIV-RNA levels compared to those receiving placebo. After 24 weeks of treatment, 53% of the patients achieved HIV RNA suppression (Lataillade et al. 2020). Most frequent AE were nausea and the elevations in liver enzymes.

*Artesunate* (Zou et al. 2020) is a semi-synthetic artemisinin derivative approved to treat severe malaria as the first-line treatment. It is an IV injection given three times on the first 24 h of the treatment, followed by a complete oral antimalarial therapy. It has been demonstrated to lower mortality more than classical quinine treatment (Dondorp et al. 2010). Kidney failure, hemoglobinuria, and jaundice are the most common AE. *Nifurtimox* (Lascano et al. 2020) is a nitrofuran antiprotozoal to treat Chagas disease that is caused by the parasite *Trypanosoma cruzi*, is the first therapy to treat pediatric patients. Sixty days of nifurtimox treatment was reported to achieve negative seroconversion and seroreduction in 32% of the patients (Altcheh et al. 2021). Nifurtimox treatment may cause serious reactions including worsening of neurologic and mental conditions and common AE such as vomiting and abdominal pain. A pediculicide, *abametapir* was approved to be used to treat head lice in pediatric patients over 6 months of age in a lotion form. Of the subjects receiving abametapir treatment, 81.5% were lice free after 14 days (Bowles et al. 2018).

## Gastroenterology

*Lactitol* (Miller et al. 2014) is an osmotic laxative that was approved for the treatment of chronic idiopathic constipation in adults, but is mostly considered for the prevention of hepatic encephalopathy. It is in powder form that should be mixed with a beverage once a day. It has been demonstrated to increase fecal volume, moisture content, and bowel movement frequency (Cheng et al. 2019). Its most common AE are upper respiratory infections, gassiness, diarrhea, and increased creatinine phosphokinase levels. The dopamine D_2_ receptor antagonist *amisulpride* (Zhang et al. 2020) was approved to prevent or treat postoperative nausea and vomiting in adults (Habib et al. 2019). Increased prolactin levels, chills, hypokalemia, and hypotension are the most frequent AE. In Europe and Australia, it was also approved as an antipsychotic drug.

## Dermatology

*Tirbanibulin*, a microtubule inhibitor is used for actinic keratosis that is caused by long-term sun exposure or tanning and may progress to skin cancer if not treated. It is an ointment applied on the affected skin once a day for 5 days. After 5 days of treatment, 44% of the patients treated with tirbanibulin and 5% of those receiving vehicle experienced a complete clearance (Blauvelt et al. 2021). The most frequent side effects were local skin reactions, application site pruritus, and application site pain. *Clascoterone* (Alkhodaidi et al. 2021) is an androgen receptor inhibitor that was approved to treat acne vulgaris in patients over 12 years of age. It is in cream form that is applied to the affected area twice a day. Clascoterone-receiving patients experienced a significant reduction in inflammatory and noninflammatory lesions after 12 weeks of treatment (Hebert et al. 2020). Reddening or itching of the skin may be observed as AE. Both therapies were approved as first-in-class medication.

## Other

An insulin-like growth factor-1 receptor inhibitor, *teprotumumab-trbw* is used to treat thyroid eye disease also known as Graves’ orbitopathy or thyroid-associated ophthalmopathy. It is a rare autoimmune disease and characterized by proptosis due to the inflamed muscle and fat tissue. It is the first therapy approved for this disease. Six months of treatment has been reported to result in 71–83% reduction in proptosis (Douglas et al. 2020). The most frequent AE are muscle spasm, hair loss, fatigue, and hyperglycemia.

The β_3_-adrenoceptor agonist *vibegron* was approved for the treatment of the overactive bladder syndrome, following earlier approval in Japan. In an international phase III study, 75 mg vibegron was compared to both placebo and to 4 mg tolterodine extended release in a total of 1518 patients (Staskin et al. 2020). Vibegron improved the number of daily micturitions and incontinence episodes by 1.8 and 2.0, respectively, as compared to 1.3 and 1.4 for placebo and 1.6 and 1.8 for tolterodine. Treatment discontinuation was observed in 1.7%, 1.1%, and 3.3% of patients receiving vibegron, placebo, and tolterodine, respectively. Headache, urinary and upper respiratory tract infections, and common cold were among the most common side effects associated with vibegron treatment. After the approval of mirabegron in 2012, vibegron is the second β_3_-adrenoceptor agonist entering the market for this indication. Presently, available data are insufficient to determine whether its efficacy and/or safety differ substantially from that of mirabegron.

*Berotralstat* is a plasma kallikrein inhibitor (Ohsawa et al. 2020) and was approved to treat hereditary angioedema with repeated episodes of severe swelling in various body parts as the first oral, once-daily therapy. A reduction in attack rate was observed in the patients receiving berotralstat compared with those receiving placebo (Zuraw et al. 2020). The most common AE reported in that clinical trial were abdominal pain, vomiting, diarrhea, and back pain. *Lonafarnib* (Young et al. 2013) is a farnesyltransferase inhibitor and used to reduce the risk of death due to Hutchinson-Gilford Progeria Syndrome (HPGS) and Progeroid Laminopathies. HPGS is a rare inherited disorder characterized by premature aging. It is caused by LMNA gene mutation that encodes lamins which are inner nuclear membrane proteins that play crucial roles in nuclear function (Guilbert et al. 2020). Mutation in this gene causes lamin accumulation in the nucleus and thereby replicative senescence (Dhillon 2021). Farnesyltransferase inhibitors block this accumulation and improve disease status. Lonafarnib treatment has been shown to increase the lifespan by 2.5 years (Dhillon 2021). In a clinical trial in which primary outcome success was defined by improved per-patient rate of weight gain and carotid artery echo density, 71% of the patients succeeded (Gordon et al. 2016). Along with serious AE such as kidney, eye, and fertility problems, common AE including vomiting, diarrhea, and decreased appetite may also been seen. Lonafarnib is the first approved therapy for these rare disorders with genetic mutations for the pediatric patients over 1 year of age.

## Diagnostic agents

Various radioactive diagnostic agents were approved for cancer detection. *Fluoroestradiol F18* (Katzenellenbogen 2021) was approved to detect estrogen receptor-positive lesions visually in patients with breast cancer. Diagnostic accuracy and safety of fluoroestradiol F18 and estrogen receptor status were confirmed in clinical trials (Chae et al. 2019). Injection site pain and taste change may be seen following its use. *Gallium 68 PSMA-11* (Hofman et al. 2021) is the first FDA-approved drug to image prostate-specific membrane antigen-positive lesions in men with prostate cancer. The safety and efficacy of Ga 68 PSMA-11 for detecting local vs. systemic disease were confirmed in distinct clinical trials (Ceci et al. 2019). Nausea, diarrhea, and dizziness are the most common AE associated with its use. For the detection of somatostatin receptor-positive neuro-endocrine tumors, *copper Cu 64 dotatate* (Hicks et al. 2019) was approved for the first time in the USA which have been reported to be a safe technique clinically (Delpassand et al. 2020). Nausea and flashing may be observed. *Flortaucipir F18* (Timmers et al. 2019) was approved as the first radioactive diagnostic agent to detect distinctive characteristics of Alzheimer’s disease, i.e., the density and distribution of aggregated tau neurofibrillary tangles that are a marker of Alzheimer’s disease. Various clinical trials have reported that flortaucipir has a potential for staging phases of the disease (Wang et al. 2016). Headache and injection site pain may be seen after its use. All these agents are for IV injection applied before positron emission tomography imaging.

## General trends and conclusions

COVID*-19* was the biggest, newly emerging, global healthcare problem of 2020 and also affected the work of regulatory authorities. Nonetheless, the FDA approved 53 novel active pharmaceutical ingredients in 2020. Among them, 6 therapeutics were the first for a specific indication including COVID-19, cholangiocarcinoma, epithelioid sarcoma, Ebola, premature aging, obesity due to enzyme deficiency, and thyroid eye disease. On the other hand, cancer therapies dominated the list, followed by neurology and anti-infective drugs (Mullard 2021). Given the typical time needed to discover and develop new drugs, it is not surprising that the only approved drug for the treatment of COVID-19 resulted from a repurposing and was approved under an emergency use authorization. This approval as well as that of SARS-CoV-2 vaccines in the USA and other jurisdictions shows that regulatory authorities can act swiftly in times of crisis without undue sacrifices on the side of diligent evaluation of new drugs.

Fifteen of the 53 novel treatments are approved as first in class that use a unique mechanism of action to treat a medical condition. In addition, 31 FDA-approved products were approved for rare or “orphan” diseases in 2020. The FDA has developed four approaches to expedite drug development process. Fast Track facilitates the development of therapies for serious conditions. Seventeen products have been designated as Fast Track in 2020. Breakthrough therapy status accelerates to develop therapies for serious condition of which preliminary clinical data provides evidence, and 22 drugs approved this year were been designated as breakthrough by the FDA. On the other hand, 30 of the 53 novel drugs received a priority review which is expected to provide a critical advance and determines a target to follow the product for 6 months, rather than 10 months. Ultimately, FDA’s accelerated approval program permits an approval of drugs for serious condition, and 12 novel drugs are approved under the accelerated approval program.

Among the newly approved drugs in 2020, small molecules are the leading drug type with 38 new drugs and of those, eight products have kinase-inhibiting properties. Moreover, 12 monoclonal antibody-based therapies are approved suggesting a prominent role for this technology. Other than antibodies, there is another protein-based therapy which is a hormone: somapacitan. Moreover, 2 gene therapies were approved, the antisense oligonucleotid, viltolarsen and the siRNA, lumasiran.
